# MdNup54 Interactions With MdHSP70 Involved in Flowering in Apple

**DOI:** 10.3389/fpls.2022.903808

**Published:** 2022-07-05

**Authors:** Chenguang Zhang, XIaoshuang Zhang, Bo Cheng, Junkai Wu, Libin Zhang, Xiao Xiao, Dong Zhang, Caiping Zhao, Na An, Mingyu Han, Libo Xing

**Affiliations:** ^1^Hebei Key Laboratory of Horticultural Germplasm Excavation and Innovative Utilization, College of Horticulture Technology, Hebei Normal University of Science and Technology, Changli, China; ^2^College of Horticulture, Northwest A&F University, Yangling, China

**Keywords:** apple, MdNup54, interaction, MdHSP70, flowering

## Abstract

Flowering-related problems in “Fuji” apple have severely restricted the development of China’s apple industry. Nuclear pore complexes (NPCs) control nucleoplasmic transport and play an important role in the regulation of plant growth and development. However, the effects of NPCs on apple flowering have not been reported. Here, we analysed the expression and function of *MdNup54*, a component of apple NPC. *MdNup54* expression was the highest in flower buds and maintained during 30–70 days after flowering. *MdNup54*-overexpressing (OE) *Arabidopsis* lines displayed significantly earlier flowering than that of the wild type. We further confirmed that MdNup54 interacts with MdHSP70, MdMYB11, and MdKNAT4/6. Consistent with these observations, flowering time of *MdHSP70*-OE *Arabidopsis* lines was also significantly earlier. Therefore, our findings suggest a possible interaction of MdNup54 with MdHSP70 to mediate its nuclear and cytoplasmic transport and to regulate apple flowering. The results enhance the understanding of the flowering mechanism in apple and propose a novel strategy to study nucleoporins.

## Introduction

The nucleus is the main site for the regulation of genetic processes and metabolism in eukaryotes. The nuclear pore complex (NPC) constitutes the nuclear pore, which is the main channel for communication between the nucleus and cytoplasm, and it controls the flow of macromolecules such as RNA and proteins in and out of the nucleus. NPCs are essential for the maintenance of normal cellular functions and play a vital role in growth and development (D’Angelo and Hetzer, 2008; Zhang A. et al., 2020). NPCs are composed of various nucleoporins (Nups). Till date, more than 30 Nups have been identified in *Arabidopsis* and apple (Tamura et al., 2010; Zhang C. et al., 2020).

Nups play an important role in plant growth, development, and stress response pathways (Xu and Meier, 2008; Greco et al., 2012; Yang et al., 2017). For example, the resistance of *nup96* mutants against infection by pathogens such as *Peronospora parasitica 4* (RPP4), *Pseudomonas syringae pv. maculicola* (RPM1), and *Pseudomonas syringae 4* (RPS4) was decreased (Zhang and Li, 2005). Nucleoporin Nup160 and Seh1 regulate the levels of Enhanced disease susceptibility1 (EDS1) and are involved in plant immunity (Roth and Wiermer, 2012). Moreover, Arabidopsis *Nup62*, *Nup96*, *Nup160*, and *NUCLEAR PORE ANCHOR* (*TPR/NUA*) are involved in auxin signalling pathways (Parry et al., 2006; Boeglin et al., 2016), and HIGH EXPRESSION OF OSMOTICALLY RESPONSIVE GENES1 (HOS1) and CONSTITUTIVE EXPRESSOR OF PATHOGENESIS-RELATED GENES 5 (CPR5) regulate ethylene signalling (Lee and Seo, 2015; Wang et al., 2017). Furthermore, HOS1 and Nup160 are involved in regulating low-temperature stress response (Dong C. et al., 2006; Dong C. H. et al., 2006), while CPR5 is involved in high-temperature stress response (Wang et al., 2012). In addition, Nup85 mediates the regulation of plant salt tolerance by interacting with MEDIATOR SUBUNIT 18 (MED18) (Zhu et al., 2017).

Nups play an important role in regulating flowering. Nup136 is a plant-specific nucleopore protein, and its mutation leads to a significant acceleration of flowering in *Arabidopsis* (Tamura et al., 2010). HOS1 regulates the binding of certain nuclear genes to *FLOWERING LOCUS C* (*FLC*) chromatin at low temperatures and reduces the transcriptional inhibition of *FLC* by HISTONE DEACETYLASE 6 (HDA6), thereby inhibiting plant flowering (Jung et al., 2013). Compared with the wild type (WT), the *nup54*, *nup58*, *nup62*, and *nup160* deletion mutants show significantly early flowering, whereas the *nup85*, *nup98A*, and *seh1* deletion mutants show no significant difference in flowering time (Parry, 2014). It was further found that *nup98A* and *nup98B* single mutants show no obvious abnormal phenotype compared to WT, while the *nup98A*/*nup98B* double mutants show an early flowering phenotype (Jiang et al., 2019). In *Arabidopsis thaliana*, Nup96 and HOS1 mutually enhance the stability of one another. HOS1 binds to and degrades CONSTANS (CO), thereby promoting *FLC* transcription and leading to delayed flowering, while Nup96 maintains this regulatory pathway to control flowering time (Lazaro et al., 2015; Cheng et al., 2020).

Heat shock proteins (HSPs) are a class of chaperones that play a crucial role in protein folding, assembly, translocation, and degradation. They stabilise proteins and cell membranes and assist in protein refolding under stress conditions (Wang et al., 2004). According to their molecular weight, HSPs are divided into five subfamilies: HSP100, HSP90, HSP70, HSP60, and small HSP (Vierling, 1991). HSPs were first studied for their involvement in heat tolerance in plants (Lindquist, 1986). In *Arabidopsis*, HSP70 inhibits the activities of HEAT SHOCK FACTOR A1 and B1 (HSFA1 and HSFB1), whereas HSP90 stimulates the DNA-binding activity of HSFB1. HSP90 is also involved in regulating the rate of synthesis of HSFA2 by controlling the degradation of *HSFA2* transcripts, thus affecting the abundance of HSFA2 and HSFB1 (Hahn et al., 2011). In cotton, the overexpression of *AsHSP70* in plants significantly enhances heat tolerance (Batcho et al., 2021).

In addition to heat tolerance, *HSPs* have been reported to participate in other stress response pathways such as salt and drought stress response (Zou et al., 2012; Wang et al., 2019; He et al., 2021). Further, the function of *HSPs* in flowering has been reported. *HSP90* plays an important role in floral induction and flower development in *Arabidopsis* (Margaritopoulou et al., 2016). *HSP101* promotes flowering under non-stressed conditions, in association with *FLC* and *SHORT VEGETATIVE PHASE (SVP)* (Qin et al., 2021).

Apple (*Malus domestica*) is a globally important fruit grown predominantly in temperate regions. The apple variety “Fuji” covers the largest cultivated area in China. However, difficulty in flowering and alternate bearing has restricted the development of the apple industry in China. Therefore, it is particularly important to strengthen research on floral transition in apple. In this study, we analysed the expression of *MdNup54* and developed early flowering MdNup54-overexpression (OE) lines in *A. thaliana* for functional characterisation. In addition, four interacting proteins [MdMYB11, HOMEOBOX PROTEIN KNOTTED-1 LIKE 4/6 (MdKNAT4/6), and MdHSP70] of MdNup54 were screened and validated. *MdHSP70*-OE *Arabidopsis* was shown to promote early flowering. Our findings provide a reference for further research on the various functions of *MdNup54*.

## Results

### Sequence and Expression Analysis of *MdNup54*

**FIGURE 1:**
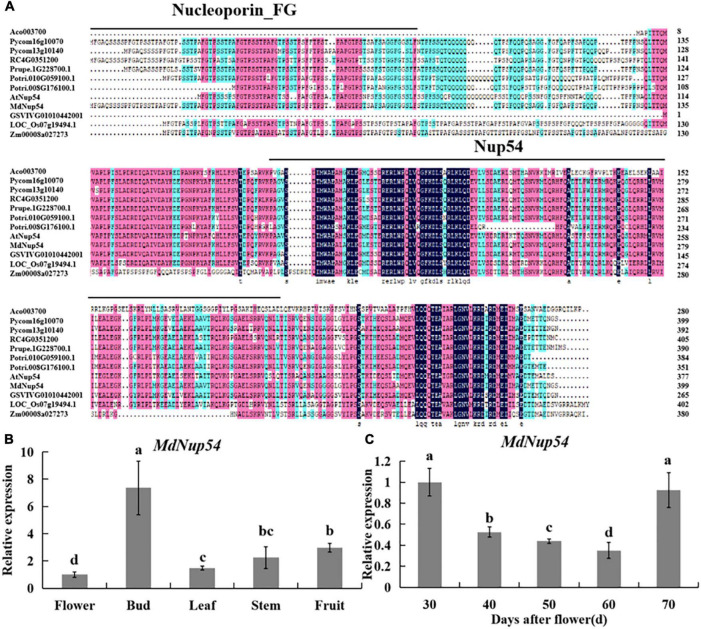
Bioinformatics and expression analysis of MdNup54. **(A)** The conservative domain of *Nup54* in 10 plant species (*Arabidopsis thaliana*, *Malus domestica*, *Populus trichocarpa*, *Oryza sativa*, *Rosa chinensis*, *Pyrus communis*, *Ananas comosus*, *Vitis vinifera*, *Zea mays*, and *Prunus persica*). **(B,C)** Analyses of *MdNup54* expression levels **(B)** in diverse “Nagafu No. 2” apple tissues and **(C)** in different flower bud developmental stages of “Nagafu No. 2.”

Multiple sequence alignment of *Nup54* homologs of 10 plant species (*A. thaliana*, *Malus domestica*, *Populus trichocarpa*, *Oryza sativa*, *Rosa chinensis*, *Pyrus communis*, *Ananas comosus*, *Vitis vinifera*, *Zea mays*, and *Prunus persica*) revealed a universally conserved Nup54 domain, while eight of them also showed a conserved Nucleoporin_FG domain (Figure 1A), suggesting that Nup54 is conserved across these plants. Tissue-specific expression analysis showed that the expression level of *MdNup54* was the highest in flower buds and the lowest in mature flowers (Figure 1B). Moreover, the expression of *MdNup54* in the terminal bud of short shoots increased during the initial stages of development, peaked between 30 and 70 days after flowering, and showed a decreasing trend thereafter (Figure 1C). These results indicate that *MdNup54* is highly expressed during the physiological differentiation of floral buds, suggesting a possible involvement in floral development in apple.

### Subcellular Localisation of MdNup54

**FIGURE 2:**
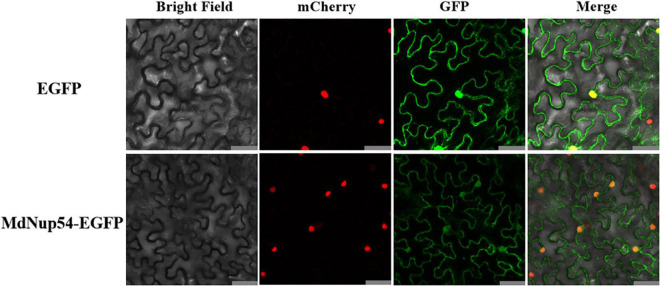
Subcellular localisation of MdNup54. The upper panel shows 35S:EGFP, and the lower panel shows 35S:MdNup54-EGFP. Bar = 50 μm.

To analyse the subcellular localisation of MdNup54, the 35S:*MdNup54-GFP* construct was introduced into tobacco leaves. Tobacco leaves infiltrated with the empty vector were used as the control, which showed an instantaneous conversion of 35S:*GFP*. In tobacco leaves expressing 35S:*MdNup54-GFP*, the GFP signal was observed specifically in the nucleus and cytoplasm, whereas it was detected throughout the cells of the control tobacco leaves (Figure 2). These observations indicate that apple MdNup54 is localised in both nucleus and cytoplasm.

### Overexpression of *MdNup54* Promotes Early Flowering

**FIGURE 3:**
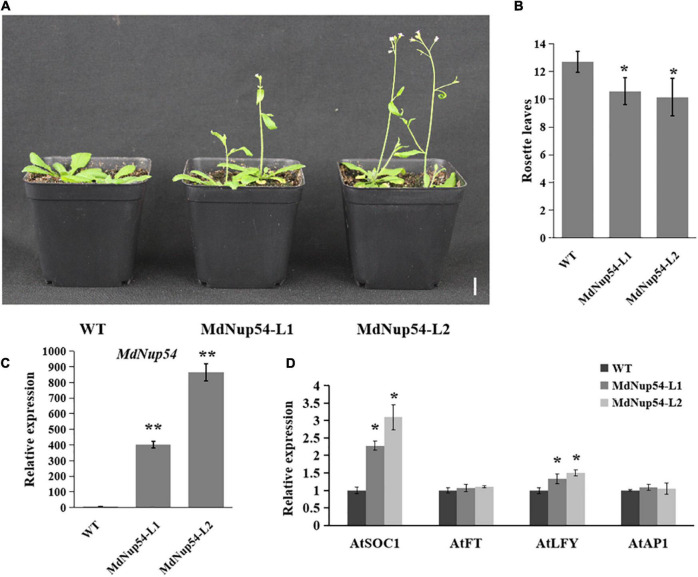
MdNup54 promotes flowering in Arabidopsis. **(A)** Phenotype of the *MdNup54*-OE Arabidopsis lines for flowering time. Bar = 2 cm. **(B)** Statistical analysis of rosette leaves of *Arabidopsis thaliana* during bolting (*n* = 15 plants per line). (*P < 0.05). **(C)** qRT-PCR analysis of *MdNup54* expression in Arabidopsis samples. (**P < 0.01). **(D)** Relative expression levels of flowering genes (*AtFT*, *AtLFY*, *AtSOC1*, and *AtAP1*) in WT and *MdNup54*-OE lines. (*P < 0.05).

For the functional characterisation of *MdNup54*, *A. thaliana* was transformed with the *MdNup54* overexpression construct to obtained two transgenic lines (*MdNup54*-L1 and *MdNup54*-L2), and their flowering phenotypes were verified. Figure 3A shows that both *MdNup54*-OE *A. thaliana* lines flowered considerably earlier than WT plants did. The *MdNup54*-OE lines had fewer rosette leaves than those of WT (Figure 3B). Genotyping and expression analysis confirmed the presence of *MdNup54* in transgenic plants (Figure 3C). Consistent with this, the expression levels of flowering genes *A. thaliana SUPPRESSOR OF OVEREXPRESSION OF CONSTANS 1* and *LEAFY* (*AtSOC1* and *AtLFY*) in transgenic *A. thaliana* were significantly higher than those in WT (Figure 3D). These results demonstrate that *MdNup54* promotes early flowering in plants.

### MdNup54 Interacts With MdHSP70, MdKNAT4/6, and MdMYB11

**FIGURE 4:**
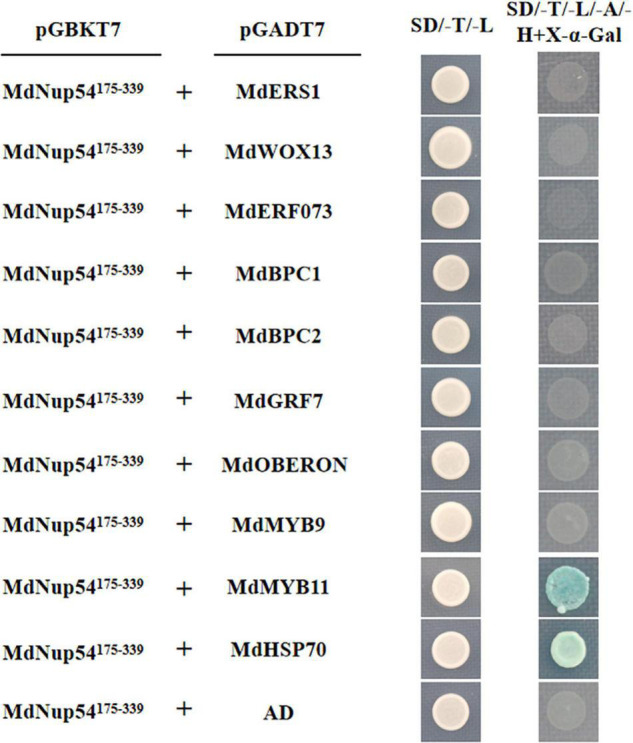
Yeast two-hybrid validation of MdNup54 interacting proteins. The *MdNup54^175– 339^* truncated sequence was cloned into pGBKT7, whereas others were cloned independently into the pGADT7 vector. Empty pGADT7 plus *MdNup54^175– 339^*-pGBKT7 was used as the control.

**FIGURE 5:**
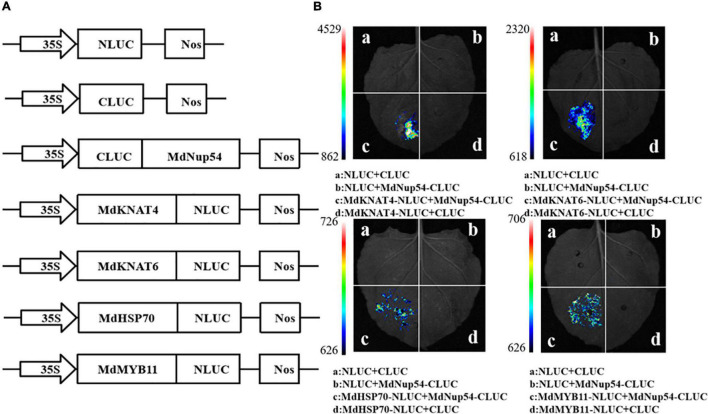
Luciferase (LUC) complementation validation of MdNup54 interacting proteins. **(A)** Carrier construction information of NLUC and CLUC. **(B)** Fluorescent signal in different combination.

To investigate the mechanism through which MdNup54 is associated with the flowering pathway, a cDNA library representing the apple flower bud was screened using a yeast two-hybrid system to identify the interacting proteins. The interactions between MdNup54 and 10 proteins were verified, and MdNup54 was found to interacte with MdHSP70 and MdMYB11 (Figure 4). In addition, since yeast interactions between MdNup54 and MdKNAT4 and MdKNAT6 have been previously reported (Zhang C. et al., 2020), the interactions between MdNup54 and these four proteins were further validated using a split-LUC complementation assay. The combination of NLUC plus CLUC, NLUC plus MdNup54-CLUC, and MdHSP70/MdKNAT4/6/MdMYB11-NLUC plus CLUC displayed no fluorescence signal, whereas the combination of MdHSP70/MdKNAT4/6/MdMYB11-NLUC plus NUP54-CLUC showed obvious fluorescence signals (Figures 5A,B). These results confirm the interaction of MdNup54 with MdHSP70, MdKNAT4/6, and MdMYB11.

### Subcellular Localisation of MdHSP70 and MdKNAT4/6

**FIGURE 6:**
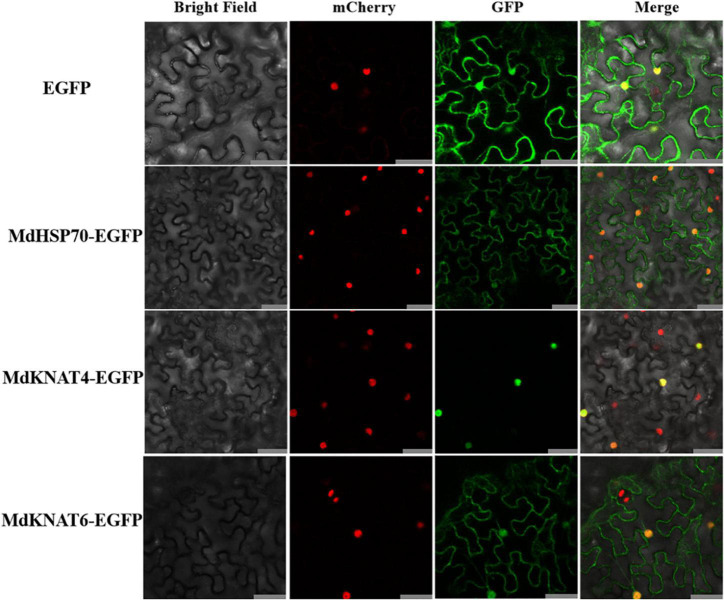
Subcellular localisation of MdHSP70 and MdKNAT4/6. The upper panel shows 35S:EGFP, the second is 35S:MdHSP70-EGFP, the third is 35S:MdKNAT4-EGFP, and the lower panel shows 35S: MdKNAT6-EGFP. Bar = 50 μm.

The subcellular localisation of the four interacting partners of MdNup54 identified from the previous experiment were analysed. Since the nuclear localisation of MdMYB11 has already been reported (An et al., 2015), the subcellular localisation of MdHSP70 and MdKNAT4/6 was studied by introducing 35S:*MdHSP70-GFP* and 35S:*MdKNAT4/6-GFP* into tobacco leaves. Tobacco leaves infiltrated with empty vector, displaying an instantaneous expression of 35S:*GFP*, were used as controls. In the tobacco leaves expressing *MdHSP70-GFP* and *MdKNAT6-GFP*, fluorescence signals were observed in the nucleus and cytoplasm, and MdKNAT4-GFP was observed only in the nucleus. In contrast, the fluorescence signal was detected throughout the control tobacco leaf cells expressing 35S:*GFP* (Figure 6). These results indicate that MdHSP70 and MdKNAT6 were localised in the nucleus and cytoplasm, whereas MdKNAT4 was localised in the nucleus.

### Overexpression of *MdHSP70* Promotes Early Flowering

**FIGURE 7:**
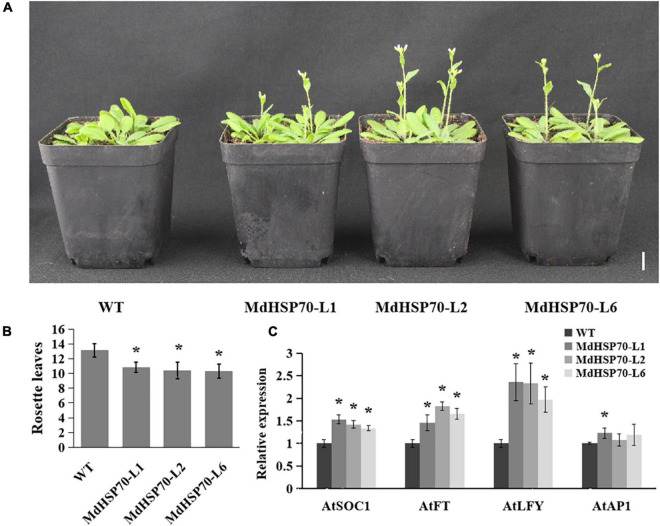
MdHSP70 promotes flowering in Arabidopsis. **(A)** Phenotype of the *MdHSP70*-OE Arabidopsis lines for flowering time. Bar = 2 cm. **(B)** Statistical analysis of rosette leaves of *Arabidopsis thaliana* during bolting (*n* = 15 plants per line). (*P < 0.05). **(C)** Relative expression levels of flowering genes (*AtFT*, *AtLFY*, *AtSOC1*, and *AtAP1*) in WT and *MdNup54*-OE lines. (*P < 0.05).

*MdHSP70*-OE transgenic *A. thaliana* lines were developed, and three lines (lines 1, 2, and 6) were selected to analyse flowering phenotypes. Genotyping and qRT-PCR-based expression analysis confirmed the presence of *MdHSP70* in the transgenic plants. Figure 7A shows that all *MdHSP70*-OE plants flowered considerably earlier than the WT plants, and the number of rosette leaves was markedly lower than that of WT plants (Figure 7B). The expression of flowering genes (*AtSOC1*, *AtFT*, and *AtLFY*) were also increased in transgenic plants in comparison to WT (Figure 7C). These results clearly demonstrated that *MdHSP70* promotes early flowering in plants.

## Discussion

Nups play an important role in the regulation of crucial life processes in plants such as flowering. In the gene regulatory network of flowering, HOS1 not only weakens the transcriptional inhibition of *FLC* by HDA6 but also directly binds and degrades CO, thus promoting the expression of *FLC* and delaying *Arabidopsis* flowering (Jung et al., 2013). Moreover, Nup96 and HOS1 directly interact with each other to maintain the respective protein stability and flowering inhibition (Lazaro et al., 2015; Cheng et al., 2020). Mutations in *Nup54*, *Nup58*, *Nup62*, *Nup136*, and *Nup160* significantly promote early flowering in *Arabidopsis* (Tamura et al., 2010; Parry, 2014). In this study, *MdNup54* was highly expressed during the physiological differentiation of apple flower buds, and its heterologous overexpression in *Arabidopsis* significantly promoted early flowering. This suggested that the phenotype of *MdNup54*-OE in *Arabidopsis* was similar to the phenotype of the *atnup54* deletion mutant in terms of flowering time. Interestingly, certain Nup overexpression and deletion mutants have been shown to display similar phenotypes, consistent with our findings. For instance, *Arabidopsis HOS1*-OE plants and *hos1* deletion mutant both suppressed the response against cold stress (Ishitani et al., 1998; Dong C. et al., 2006). Moreover, *AtNup62* showed a similar pattern in auxin response (Boeglin et al., 2016). These observations indicate that in case of nucleoproteins, overexpression does not result in gain-of-function, and instead a functional loss is noted yielding a phenotype similar to the mutant. Therefore, it is not surprising that the overexpression of *MdNup54* in this study also resulted in loss-of-function. Nonetheless, the findings confirmed the involvement of *MdNup54* in the flowering pathway.

To elaborate the mechanism of action of MdNup54, the interactions between MdNup54 and MdMYB11, MdKNAT4/6, and MdHSP70 were screened and confirmed. Nups play an important role in controlling the entry and exit of molecules in the nucleus (Zhang A. et al., 2020), and Nup54 plays a key role in nuclear transport owing to its localisation in the central part of the nuclear pore. Thus, apple MdNup54 may control the transport of MdMYB11, MdKNAT4/6, and MdHSP70 and participate in the corresponding biological processes. *MYB11* is induced by methyl jasmonate (MeJA), and the overexpression of *MYB11* significantly promotes the accumulation of anthocyanins and proanthocyanidins in apple calli (An et al., 2015). Furthermore, members of the *KNOX* family participate in plant hormone signalling (Jasinski et al., 2005; Sakamoto et al., 2006; Bolduc and Hake, 2009) and flower development (Khan et al., 2015). The *MdKNAT4* and *MdKNAT6* genes in apple are homologous to *AtKNAT4* and *AtKNAT6* genes in *Arabidopsis*, respectively. Previous studies have confirmed that *AtKNAT4* affects seed dormancy (Chai et al., 2016), whereas *AtKNAT6* plays an important role in maintaining meristem integrity and flowering (Khan et al., 2015). Moreover, *MdHSP70* belongs to the *HSP* family and plays an important role in high-temperature stress tolerance (Hahn et al., 2011). Combined with these results, it is speculated that MdNup54 may be involved in colouration, flowering, seed germination, and response to high-temperature stress in apples.

Heat shock proteins have been reported to be involved in flowering (Margaritopoulou et al., 2016; Qin et al., 2021). HSP90 directly interacts with LFY, SOC1, and AGAMOUS-LIKE 24 (AGL24) to regulate flower differentiation, and the RNAi lines with suppressed *HSP90* expression display severely impeded flowering (Margaritopoulou et al., 2016). The *athsp101*-knockout and *AtHSP101*-OE lines show delayed and advanced flowering, respectively. In addition, the late flowering phenotype can be restored by rescuing *AtHSP101*. Moreover, the promotion of flowering by *AtHSP101* was found to be dependent on FLC and SVP (Qin et al., 2021). The *MdHSP70*-OE *Arabidopsis* lines obtained in this study significantly promoted early flowering. This suggested a possible role of *MdHSP70* in apple flowering. As both *MdNup54* and *MdHSP70* are involved in the flowering process, we speculate that the mechanism through which MdNup54 influences flowering in apple involves the control of nuclear and cytoplasmic transport of HSP70.

Overall, the present study provides evidence regarding the role of a member of the NPC *MdNup54* in regulating floral transition in apple. The *MdNup54* gene is highly expressed in floral buds and during floral transition, and its heterologous overexpression in *A. thaliana* leads to early flowering. In addition, the MdNup54 protein was localised to both the nucleus and cytoplasm. Furthermore, the findings demonstrate that this function in floral transition is most likely mediated by interaction with several crucial genes involved in various developmental and stress response pathways, i.e., MdNup54, MdMYB11, MdKNAT4/6, and MdHSP70. *MdHSP70* was also shown to accelerate flowering in *A. thaliana*.

## Materials and Methods

### Plant Materials and Growth Conditions

The plant materials were 6-year-old apple trees (“Fuji”/T337/*Malus robusta* Rehd.) growing in the experimental orchard of the Horticulture College of Northwest A&F University (108°04′E, 34°16′N). The new shoots (2–3 mm in diameter) near the tips, fully expanded leaves near buds, flower buds, blooming flowers, and young fruit were collected for tissue specific expression analysis. And terminal bud of short shoots at 30, 40, 50, 60, and 70 days after flowering were collected. All the samples were immediately frozen in liquid nitrogen and stored at −80°C for later use.

Arabidopsis plants (“Columbia”) growed under long-day conditions (16 h-light/8 h-dark) at 22°C. Measurements of flowering times were carried out when the plants were 4 weeks old after germination.

### Protein Alignment Analysis

A protein sequence alignment of *Nup54* from 10 plant species was performed using DNAMAN software. And the software was downloaded from LynnonBiosoft.^1^

### RNA Extraction and Quantitative Real-Time PCR Analysis

The different tissues of apple trees (new shoots, leaves, flower buds, flowers, and young fruit), Arabidopsis seedlings leaves, and apple seedlings leaves samples were ground to powders in mortars under frozen conditions. Then, 100 milligram (mg) from them were taken, respectively, for total RNA extracting by using an RNA Plant Plus Reagent Kit (TIANGEN, Beijing, China) according to the manufacturer’s instructions. The RNA was used as the template to synthesise cDNA with a PrimeScript RT Reagent Kit (Takara, Shiga, Japan). The qRT-PCR analysis was conducted on a StepOnePlus Real-Time PCR System (Thermo Fisher Scientific, United States). The reaction solution contained 10 μL SYBR Green I Master Mix (CWBIO, Beijing, China), 0.5 μmol⋅L^–1^ primers (SANGON BIOTECH, Shanghai, China) (Supplementary Table 1), and 1 μL of each 1:5 diluted cDNA as a template in a total volume of 20 μL. The PCR programme was as follows: 95°C for 3 min; 40 cycles of 94°C for 15 s, 60°C for 20 s, and 72°C for 15 s. Apple *MdActin* (MD04G1127400) and Arabidopsis *AtActin* (AT2G37620) were used as reference genes, and the sequences can be found in GDR^2^ and TAIR^3^ database, respectively. All the samples were analysed with three biological replicates, each comprising three technical replicates. Relative gene expression levels were calculated in accordance with the 2^–ΔΔ*Ct*^ method (Livak and Schmittgen, 2001).

### Subcellular Localisation

The open reading frames (ORFs) of the *MdNup54*, *MdHSP70*, and *MdKNAT4/6* genes were inserted independently into the pCAMBIA2300-EGFP vector to generate the 35S:*MdNup54*-EGFP, 35S:*MdHSP70*-EGFP, and 35S:*MdKNAT4/6-EGFP* recombinant plasmids, respectively. These recombinant plasmids were inserted independently into *Agrobacterium tumefaciens* strain GV3101 cells. Then they were infiltrated into tobacco leaves. GV3101 cells containing the pCAMBIA2300-EGFP vector (35S:*EGFP*) served as the control. After an additional 3 days of growth in the dark, green fluorescent protein (GFP) signals in transformed tobacco leaves were detected using a Leica TCS SP8 SR Laser Scanning Confocal Microscope (Leica, Germany). The primers used are shown in Supplementary Table 2.

### Genetic Transformation

The methods for transgenic *Arabidopsis thaliana* were according to published papers (Clough and Bent, 1998). The transgenic Arabidopsis lines were selected on MS plates supplemented with 50 mg⋅L^–1^kanamycin.

### Yeast Two-Hybrid Assay

The *MdNup54*^175–339^ truncated sequence was cloned into the pGBKT7 vector. The other ORFs were cloned individually into the pGADT7 vector. The recombinant plasmids were inserted into Gold Yeast Two-Hybrid cells, which were then grown on a selective medium. The primers used are shown in Supplementary Table 2.

### Split Luciferase Complementation

The full-length *MdHSP70, MdKNAT4/6*, and *MdMYB11* coding sequences were cloned independently into the NLUC vector, while *MdNup54* was cloned into the CLUC vector. The split-LUC complementation assay was performed with tobacco leaves. The reconstituted LUC activity was detected in the dark using a Princeton Lumazone Pylon 2048B cooling camera (Princeton, NJ, United States). The primers used are shown in Supplementary Table 2.

### Statistical Analyses

Statistical analyses were performed using SPSS software. Asterisks (*) indicate significant differences between treatments as assessed by Student’s *t*-test at *P* < 0.05 (*) and *P* < 0.01 (^**^). Different lowercase letters above the bars indicate significant differences (*P* < 0.05, Tukey’s test).

## Data Availability Statement

The original contributions presented in the study are included in the article/Supplementary Material, further inquiries can be directed to the corresponding authors.

## Author Contributions

DZ, CPZ, NA, MH, LX, and CGZ conceived and designed the experiment. CGZ, XZ, and BC performed the experiment. JW, LZ, XX, XZ, and CGZ analysed the data. CGZ and LX wrote the manuscript. All authors contributed to the article and approved the submitted version.

## Conflict of Interest

The authors declare that the research was conducted in the absence of any commercial or financial relationships that could be construed as a potential conflict of interest.

## Publisher’s Note

All claims expressed in this article are solely those of the authors and do not necessarily represent those of their affiliated organizations, or those of the publisher, the editors and the reviewers. Any product that may be evaluated in this article, or claim that may be made by its manufacturer, is not guaranteed or endorsed by the publisher.

## Footnotes

1
https://www.lynnon.com/

2
https://www.rosaceae.org

3
https://www.arabidopsis.org

## Supplementary Material

Supplementary Table 1 Primers used for qRT-PCR.

Supplementary Table 2 Primers used for plasmid construction.

Supplementary Figure 1 Genomic PCR analyses of *MdNup54* **(a)**, *MdHSP70* **(b)** and qRT-PCR analyses of *MdHSP70* **(c)** in transgenic Arabidopsis lines.
