# Supplementary material for: MdNup54 Interactions With MdHSP70 Involved in Flowering in Apple
Source: Front Plant Sci. 2022 Jul 5;13:903808. doi: 10.3389/fpls.2022.903808 (PMC9296068; doi:10.3389/fpls.2022.903808)
Supplement: Supplementary Table 1 — Primers used for qRT-PCR. [file Data_Sheet_1.docx]

**MdNup54 interactions with MdHSP70 involved in flowering in apple**

Chenguang Zhang^1^†, XIaoshuang Zhang^1^†, Bo Cheng^2^†, Junkai Wu^1^, Libin Zhang^1^, Xiao Xiao^1^, Dong Zhang^2^, Caiping Zhao^2^, Na An^2^, Mingyu Han^2^, Libo Xing^2^*,

^1^Hebei Key Laboratory of Horticultural Germplasm Excavation and Innovative Utilization, College of Horticulture Technology, Hebei Normal University Of Science & Technology, 066600, Changli, Hebei, P. R. China

^2^College of Horticulture, Northwest A&F University, 712100 Yangling, Shaanxi, P. R. China

**†Equal contributors**

***Corresponding author:**

Libo Xing

E-mail: [libo_xing@nwsuaf.edu.cn](mailto:libo_xing@nwsuaf.edu.cn) ;

Tel.: +8615129227289;

ORCID: https://orcid.org/0000-0002-8918-7128;

Address: 3 Taicheng Road, Yangling 712100, Shaanxi, P. R. Chin

Run title: MdNup54 interactions with MdHSP70 regulate flowering

| **Table S1.** | | | |
| --- | --- | --- | --- |
| **Gene name** | **Gene ID** | **Primer sequences** | **Amplicon size(bp)** |
| *MdACTIN*(reference) | MD04G1127400 | F:CAACTCATCCGAACCTCAAACC  R:CGCTGTCCGCCATCTTCTACT | 80 |
| *MdNup54* | MD16G1117500 | F:AGAGGGCAAGGGTTTCCGACTG  R:TCCGTCTGCTGCTGTAACACCT | 249 |
| *AtActin*(reference) | AT2G37620 | F:GCGATTCCGTTGTCCTGAGGTTC  R:TTCCACCACTGAGCACAATGTTACC | 149 |
| *AtFT* | AT1G65480 | F:GGTGACTAATGGCTTGGAT  R:GGACTTGGAACATCTGGAT | 123 |
| *AtSOC1* | AT2G45660 | F:GCAACAAGCAGACAAGTG  R:CTTAGTATGCCTCAGATAACG | 183 |
| *AtLFY* | AT5G61850 | F:CCGTGAGTTCCTTCTTCAG  R:CTTCTTCGTCTAGGCAGTG | 176 |
| *AtAP1* | AT1G69120 | F:GGCAATGAGGAGGAATGAT  R:GAGCCTAGCCACTATTTATATG | 153 |

**Table S2.**

| **Gene name** | **Gene ID** | | **Primer sequences** |
| --- | --- | --- | --- |
| *MdNup54*-35S-F | MD16G1117500 | ACGAGCTCGGTACCATGTTCGGAGCTCAATCT | |
| *MdNup54*-35S-R | MD16G1117500 | CATGGTGTCGACTCTAGAGCTCCCGTTCTGTGTTG | |
| *MdKNAT4*-35S-F | MD02G1012900 | ACGAGCTCGGTACCATGGCGTTTCATCACCAG | |
| *MdKNAT4*-35S-R | MD02G1012900 | CATGGTGTCGACTCTAGACCTCTTGCGTTTGCTCTT | |
| *MdKNAT6*-35S-F | MD13G1095800 | ACGAGCTCGGTACCATGGAGGAAATGTACGGA | |
| *MdKNAT6*-35S-R | MD13G1095800 | CATGGTGTCGACTCTAGATTCATTTGTAAAGAATGG | |
| *MdHSP70*-35S-F | MD14G1024700 | ACGAGCTCGGTACCATGGCCGGAGAGAACATG | |
| *MdHSP70*-35S-R | MD14G1024700 | CATGGTGTCGACTCTAGATTTGCTACTACCAAA | |
| *MdNup54*^175-339^-pGBKT7-F | MD16G1117500 | ATGGCCATGGAGGCCGAATTCAAGCCTGCTGGTGTATCG | |
| *MdNup54*^175-339^-pGBKT7-R | MD16G1117500 | CCGCTGCAGGTCGACGGATCCGGATATGGTTAATAGG | |
| *MdKNAT4*-pGADT7-F | MD02G1012900 | GCCATGGAGGCCAGTGAATTCATGGCGTTTCATCACCAG | |
| *MdKNAT4*-pGADT7-R | MD02G1012900 | CAGCTCGAGCTCGATGGATCCCTACCTCTTGCGTTTGCTC | |
| *MdKNAT6*-pGADT7-F | MD13G1095800 | GCCATGGAGGCCAGTGAATTCATGGAGGAAATGTACGGA | |
| *MdKNAT6*-pGADT7-R | MD13G1095800 | CAGCTCGAGCTCGATGGATCTCATTCATTTGTAAAGAATG | |
| *MdMYB11*-pGADT7-F | MD09G1184000 | GCCATGGAGGCCAGTGAATTCATGGGAAGGAGTCCTTGT | |
| *MdMYB11*-pGADT7-R | MD09G1184000 | CAGCTCGAGCTCGATGGATCCTTAATTATCTACGAGCCA | |
| *MdHSP70*-pGADT7-F | MD14G1024700 | GCCATGGAGGCCAGTGAATTCATGGCCGGAGAGAACATG | |
| *MdHSP70*-pGADT7-R | MD14G1024700 | CAGCTCGAGCTCGATGGATCCTCATTTGCTACTACCAAA | |
| *MdERS1*-pGADT7-F | MD03G1292200 | GCCATGGAGGCCAGTGAATTCATGGAGTCCTGTGATTGC | |
| *MdERS1*-pGADT7-R | MD03G1292200 | CAGCTCGAGCTCGATGGATCCTCAAAGACTTCTTTGATAA | |
| *MdWOX13*-pGADT7-F | MD00G1207300 | GCCATGGAGGCCAGTGAATTCATGATGGGGGAGTGGCGG | |
| *MdWOX13*-pGADT7-R | MD00G1207300 | CAGCTCGAGCTCGATGGATCCCTACGACAACTTTTATTT | |
| *MdERF073*-pGADT7-F | MD11G1306500 | GCCATGGAGGCCAGTGAATTCATGTGCGGTGGTGCTATA | |
| *MdERF073*-pGADT7-R | MD11G1306500 | CAGCTCGAGCTCGATGGATCCTCACACGGAAGCAGGAAC | |
| *MdBPC1*-pGADT7-F | MD05G1054600 | GCCATGGAGGCCAGTGAATTCATGGACGATGGTCGGCAA | |
| *MdBPC1*-pGADT7-R | MD05G1054600 | CAGCTCGAGCTCGATGGATCCCTACTTGATCGTTATGTAG | |
| *MdBPC2*-pGADT7-F | MD10G1062100 | GCCATGGAGGCCAGTGAATTCATGGATGATGGTCGGCAA | |
| *MdBPC2*-pGADT7-R | MD10G1062100 | CAGCTCGAGCTCGATGGATCCCTACTTGATCGTTATGTA | |
| *MdGRF7*-pGADT7-F | MD17G1074900 | GCCATGGAGGCCAGTGAATTCATGTCGCCACCTGATTCT | |
| *MdGRF7*-pGADT7-R | MD17G1074900 | CAGCTCGAGCTCGATGGATCCTTACTGTGCTTCACTGGA | |
| *MdOBERON*-pGADT7-R | MD09G1122100 | GCCATGGAGGCCAGTGAATTCATGGGTACATCATCTGGT | |
| *MdOBERON*-pGADT7-R | MD09G1122100 | CAGCTCGAGCTCGATGGATCCTCAGGGATTTTTTCTGAA | |
| *MdNup54*-CLUC-F | MD16G1117500 | GATCGCCGTGTCTAGAATGTTCGGAGCTCAATCTTCG | |
| *MdNup54*-CLUC-R | MD16G1117500 | GCTTGATATCGAATTCGCTCCCGTTCTGTGTTGTT | |
| *MdMYB11*-NLUC-F | MD09G1184000 | GGCGGCCGCTCTAGAATGGGAAGGAGTCCTTGT | |
| *MdMYB11*-NLUC-R | MD09G1184000 | CAGCCCGGGGGATCCTTAATTATCTACGAGCCA | |
| *MdHSP70*-NLUC-F | MD14G1024700 | GGCGGCCGCTCTAGAATGGCCGGAGAGAACATG | |
| *MdHSP70*-NLUC-R | MD14G1024700 | CAGCCCGGGGGATCCTTTGCTACTACCAAATAT | |
| *MdKNAT4*-NLUC-F | MD02G1012900 | GGCGGCCGCTCTAGAATGGCGTTTCATCACCAG | |
| *MdKNAT4*-NLUC-R | MD02G1012900 | CAGCCCGGGGGATCCCCTCTTGCGTTTGCTCTT | |
| *MdKNAT6*-NLUC-F | MD13G1095800 | GGCGGCCGCTCTAGAATGGAGGAAATGTACGGA | |
| *MdKNAT6*-NLUC-R | MD13G1095800 | CAGCCCGGGGGATCCTTCATTTGTAAAGAATGG | |


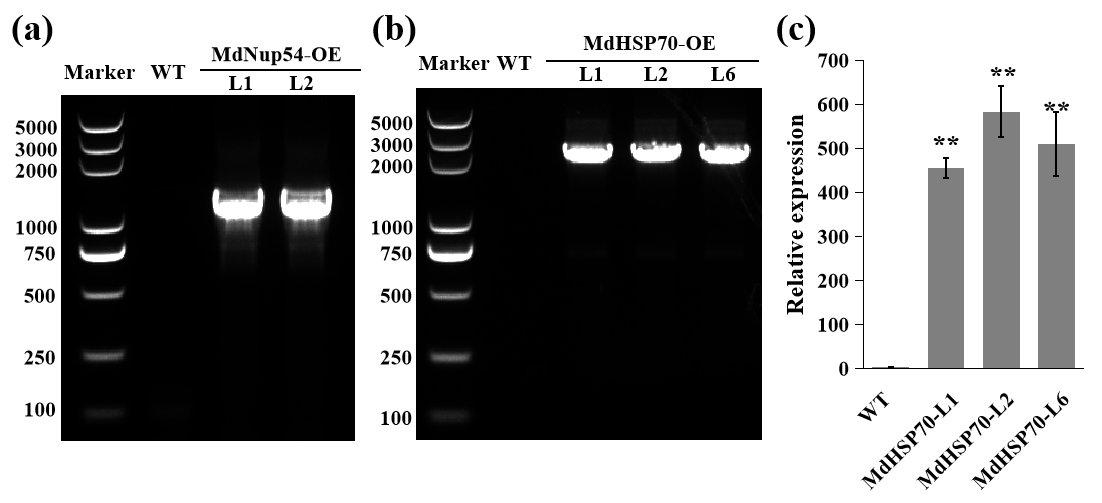


**Figure S1.**
